# Potential of automated online adaptive proton therapy to reduce margins for oesophageal cancer

**DOI:** 10.1016/j.phro.2025.100712

**Published:** 2025-01-31

**Authors:** Pascal Herbst, Camille Draguet, Ana M. Barragán-Montero, Elena Borderías Villarroel, Macarena Chocan Vera, Pieter Populaire, Karin Haustermans, Edmond Sterpin

**Affiliations:** a KU Leuven, Department of Oncology, Laboratory of Experimental Radiotherapy, Leuven, Belgium; b UCLouvain, Institut de Recherche Expérimentale et Clinique, Center of Molecular Imaging, Radiotherapy and Oncology (MIRO), Brussels, Belgium; c University Hospital Leuven, Department of Radiation Oncology, Leuven, Belgium; d RaySearch Laboratories AB, Stockholm, Sweden

**Keywords:** Proton therapy, Online adaptive, Oesophageal cancer

## Abstract

**Background and purpose:**

Proton therapy for oesophageal cancer is administered over multiple fractions, based on a single pre-treatment image. However, anatomical changes can lead to the deterioration of the treatment plan, necessitating manual replanning. To keep this within limits, increased residual margins are employed. This study aimed to evaluate the proposed automated Online Adaptive Proton Therapy (OAPT) strategies on their capability to reduce the need for manual replanning, while also exploring the possibility of margin reduction.

**Materials and methods:**

Two automated OAPT methods were examined: Automated Dose Restoration (ADR) and Automated Full Adaptation (AFA). ADR makes use of dose restoration, restoring the original dose map based on the patient’s altered anatomy. AFA adapts the contours used for plan optimization by applying a deformation field, not only correcting for density changes, but also for the relative location of organs. A comparative analysis of OAPT strategies, evaluating $D_{\text{98\%}}$ tumour coverage on 17 patients, was conducted.

**Results:**

The nominal results of non-adapted plans with 7 mm residual margins required manual replanning for 18% of the patients. ADR reduced this to 6%, while AFA eliminated the need for manual replanning. With 2 mm margins, 47% of cases required manual replanning. ADR reduced this to 18%, and AFA further reduced it to 11%.

**Conclusions:**

The proposed OAPT strategies offered a marked improvement compared to a non-adaptive approach. ADR and AFA significantly reduced the necessity for manual replanning and facilitated the reduction of residual margins, enhancing dose conformity and reducing treatment toxicity.

## Highlights

• Inadequate target coverage in 18% of non-adapted proton plans for oesophageal cancer.
• Online adaptive strategies improve target coverage, reducing manual replanning need.
• Online adaptive strategies allow reduced margins with manageable replanning levels.

## 1 Introduction

Oesophageal cancer is the seventh most diagnosed cancer and the sixth leading cause of cancer-related deaths [1]. The challenging landscape is further emphasized by a five-year survival rate of 10% in Europe [2]. The standard of care for locally advanced oesophageal cancer involves a trimodality treatment approach, including chemotherapy and radiotherapy, followed by surgical intervention [3], [4]. This treatment approach is associated with complications affecting the heart, lungs, and the gastrointestinal tract, contributing to substantial morbidity and mortality [5], [6], [7]. The high doses delivered to Organs At Risk (OAR) are predictors of complications, with lung and heart doses demonstrating a significant correlation with toxicity and postoperative complications [8], [9]. In addressing these challenges, Proton Therapy (PT) emerged as a compelling alternative, offering superior conformality and preservation of OARs [10], [11], [12], [13], [14], [15], [16], [17]. This is particularly relevant for oesophageal cancer, given the proximity of vital organs to the treatment target [11], [18].

Radiotherapy treatment plans are based on a planning image, which captures the patient’s anatomy at a single point in time. The plan is then delivered in multiple fractions. However, patients are not static, and anatomical variations and tumour shrinkage can occur during the course of treatment [19], [20]. PT is sensitive to these anatomical changes. Density alterations along the beam path disrupt the position of the Bragg peak, leading to the deterioration of the planned dose. In such cases, manual inter-fractional plan adaptation becomes necessary, resulting in the postponement of the treatment fraction. To limit the need for manual replanning, error margins are implemented [21], [22], [23]. These measures undermine the potential of PT, reducing treatment conformity and consequently increasing toxicity.

Online Adaptive Proton Therapy (OAPT) provides a way to compensate for inter-fraction changes. The objective is to automatically adapt the treatment plan to the patient’s altered anatomy for each fraction. These adjustments happen while the patient is in the treatment position, moments before treatment delivery. OAPT can improve target coverage and limit OAR dosage [24]. Furthermore, it may reduce the need for uncertainty margins [25].

Anatomical changes during treatment cause deterioration of oesophageal cancer treatment plans [26], and recent evidence emphasizes the benefits of adapting these plans [27]. Adapting treatment plans through dose restoration was shown to be effective in the treatment of head and neck cancer, lung cancer, prostate cancer, and nasopharyngeal cancer [28], [29], [30], [31]. Adaptation based on contour deformation was successfully implemented for head and neck cancers [25]. Moreover, autodelineation using deformable propagation was shown to aid the adaptive workflow for oesophageal cancer [32]. Margin reduction in adaptive magnetic resonance-guided radiotherapy for oesophageal cancer contributed to dosimetric benefits, including reduced exposure to the heart and lungs while maintaining target coverage [33], [34]. Automation of OAPT is essential for integrating these strategies into the clinical workflow [35], [36].

This study aimed to evaluate the effectiveness of proposed automated OAPT strategies in reducing the need for manual plan adaptation for oeosphageal cancer, potentially facilitating margin reduction. We conducted a comparative analysis of different adaptation strategies employing dose restoration and contour deformation.

## 2 Materials and methods

### 2.1 Patient data

A clinical trial database served as the basis for the analysis, encompassing 17 patients treated at University Hospital Leuven between 2016 and 2018. The patient data was approved by the hospital’s Institutional Ethical Review Board (S59667). These patients had locally advanced oesophageal cancer and underwent neoadjuvant chemoradiation therapy followed by surgery. Initially treated with photons, they were retrospectively planned for Intensity Modulated Proton Therapy (IMPT). All patients underwent a 4D planning Computed Tomography (pCT), followed by a 4D repeated Computed Tomography (rCT) four weeks later. The phases of the 4D scans were combined into the planning average CT and the repeated average CT. The Internal Target Volume (ITV) was constructed using the individual phase data of the 4D CT to ensure clinical target coverage in all respiratory phases. Contouring of the target and various OARs, including the lungs, heart, liver, kidney, and spinal cord, was conducted by an experienced radiation oncologist on the pCT and the rCT. This was done following the contouring guidelines provided by Thomas et al. [37]. The contours on the pCT formed the basis for the original IMPT plans. In contrast, the contours on the acquired rCT were used to evaluate the treatment plans four weeks into treatment. This dual-contouring approach enabled the assessment of the proposed OAPT strategies in response to changes in the patient’s anatomy. Anatomical changes in the selected patient cohort were primarily related to air accumulation in the stomach.

An experienced dosimetrist created the IMPT plans for pencil beam scanning, using one posterior beam at 180 degrees and one or two posterior oblique beams between 140 and 160 degrees, in accordance with the PROTECT treatment planning protocol [38]. The plans were optimized in RayStation 11B (RaySearch, Sweden) using the Monte Carlo dose engine (v5.3). Robust optimization was employed, optimizing for isotropic positional uncertainties of 7 mm and 2 mm, along with a systematic density uncertainty of 2.6%. We opted for conventional 7 mm residual margins to represent clinical practice and 2 mm residual margins to assess the possibility of margin reduction. Planning followed the PROTECT guidelines, prescribing 28 fractions of 1.8 Gy (RBE) for a total dose of 50.4 Gy (RBE) to the ITV. Biological doses were calculated using an RBE factor of 1.1.

**Fig. 1:**
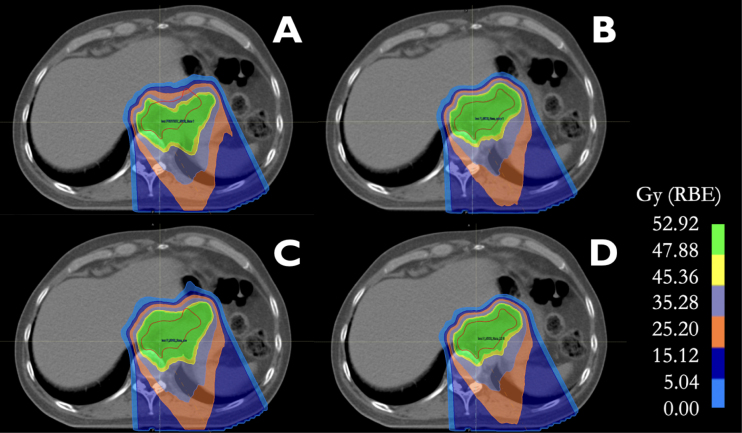
Treatment plans on rCT after anatomical change: (A) Non-Adapted, (B) Manual Contour Adaptation, (C) Automated Dose Restoration, and (D) Automated Full Adaptation, all with 7 mm residual margins. The ITV is delineated in red.

**Fig. 2:**
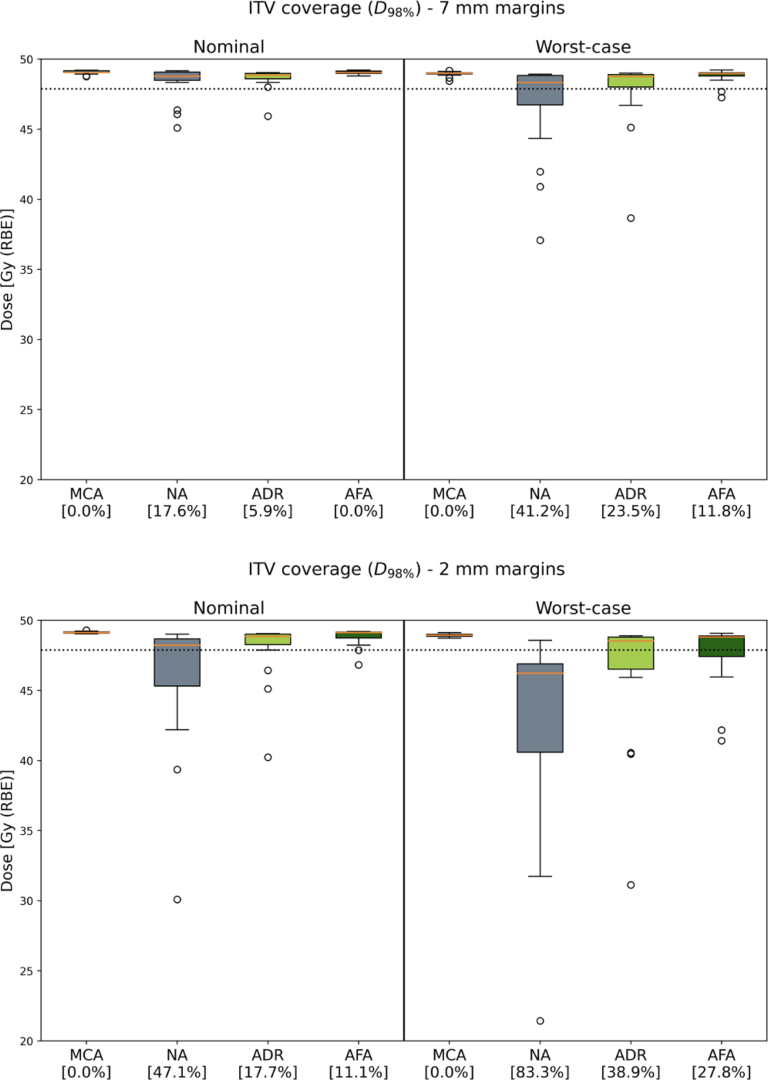
Comparison of ITV coverage ($D_{\text{98\%}}$) for Manual Contour Adaptation (MCA), No Adaptation (NA), Automated Dose Restoration (ADR) and Automated Full Adaptation (AFA). The boxes represent the interquartile range (25%–75%), with a line at the median. Outliers are shown separately. The dotted line indicates 95% of the planned dose at 47.88 Gy (RBE). The percentages represent the relative number of patients falling below this replanning threshold.

**Fig. 3:**
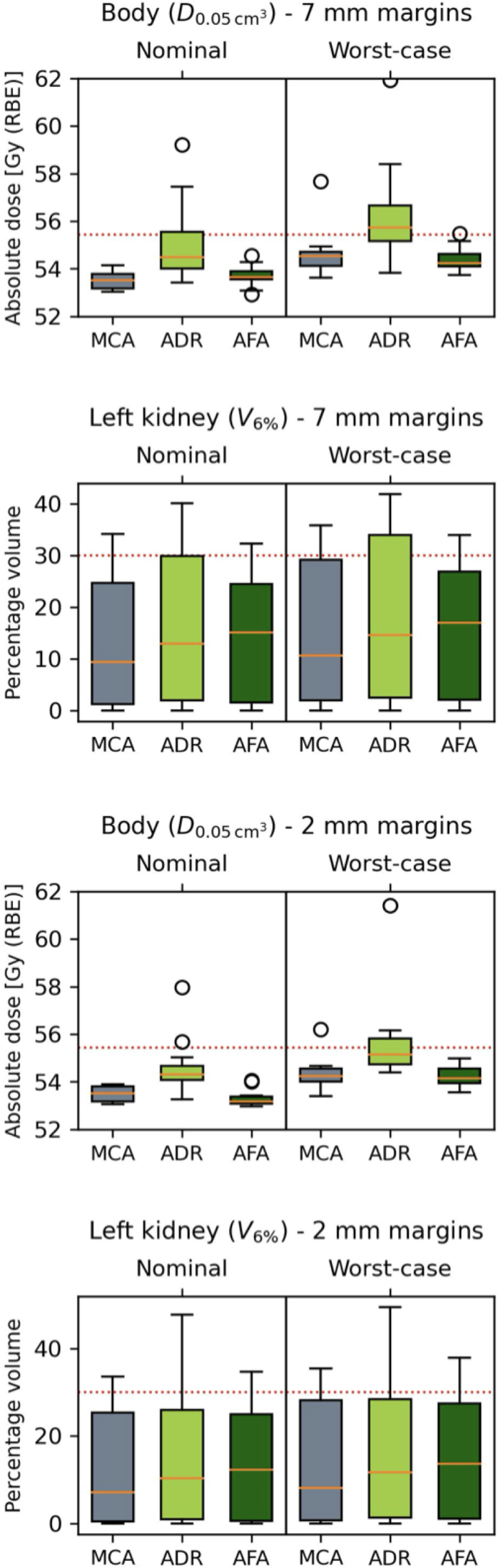
Comparison of body dosage ($D_{0.05\;{\text{cm}}^{3}}$) and left kidney dosage ($V_{6\text{\%}}$) for Manual Contour Adaptation (MCA), Automated Dose Restoration (ADR) and Automated Full Adaptation (AFA). Depicted for 2 mm and 7 mm residual margins. The boxes represent the interquartile range (25%–75%), with a line at the median. Outliers are shown separately. The red lines indicate the dose limit, as detailed in Supplementary Table 1.

### 2.2 OAPT strategies

This study explored the potential of the automated OAPT methods Automated Dose Restoration (ADR) and Automated Full Adaptation (AFA). ADR employs dose restoration to restore the original dose map on the patient’s altered anatomy, while AFA modifies the contours used for plan optimization by applying a deformation field. Both strategies were automated using Python-based scripting in RayStation 11B (RaySearch, Sweden), which allowed for seamless ’single-click’ integration.

The ADR strategy is composed of several steps. A dose map is generated from all voxels in the initial dose distribution and categorized within specific dose ranges. Using grayscale intensities within the ITV region, rigid image registration between the pCT and the rCT establishes a point-to-point correspondence. This correspondence is used to warp the dose map of the original plan onto the altered anatomy of the rCT. Re-optimization is then performed using the initial dose distribution, constraints and penalty weights of the initial clinical plan and the rCT. This approach compensates for proton range distortions by adjusting spot positions and energy layer parameters. The contours remain unadjusted to the new anatomical situation, and the plan is corrected solely for density changes along the beam path.

AFA adapts the contours used for plan optimization by applying a deformation field, correcting for density changes in the beam path, and for the relative location of organs. This strategy employs mutual information based deformable registration between the pCT and rCT to adapt the original contours to the rCT. The original contours from the pCT are deformed using a vector field obtained from the deformable registration matrix and warped onto the rCT. This facilitates the creation of an adapted treatment plan, accounting for anatomical changes. The optimization process uses the original plan’s constraints and penalty weights for the re-optimization process.

An analysis between the OAPT strategies, non-adapted plans, and Manual Contour Adaptation (MCA) plans was conducted. The AFA plans were derived through manual input from the radiation oncologist, who edited the contours based on the rCT and re-optimized the plan using the constraints and penalty weights of the initial clinical plan.

### 2.3 Robustness evaluation and statistical analysis

The plans were assessed on the rCT, with the corresponding contours delineated by a radiation oncologist serving as the ground truth. The robustness evaluation scenarios incorporated 2 mm uncertainties, in line with studies on OAPT for oesophageal cancer [26], [27], [39], [40]. For the systematic density uncertainty, we used 2.6% at a 90% confidence interval, as provided by Paganetti [21]. These uncertainties were applied to all strategies to assess a systematic online approach. A total of 28 scenarios were examined, with isocenter shifts along the axes and diagonals of the cuboid, covering both nominal and worst-case situations. We conducted a comparative analysis evaluating ITV coverage using the $D_{\text{98\%}}$. A fall below 95% of the planned dose indicated the need for manual replanning.

The clinical goals for relevant OAR metrics were derived from the PROTECT protocol (Supplementary Table 1). For body dose, the $D_{0.05\;{\text{cm}}^{3}}$ and $D_{1\;{\text{cm}}^{3}}$ were examined. The lung dose was assessed using the $D_{\text{mean}}$, $V_{20\text{\%}}$, and $V_{5\text{\%}}$, while the heart dose was evaluated using the $D_{\text{mean}}$, $V_{25\text{\%}}$, and $V_{40\text{\%}}$. The liver dose was assessed with the $V_{30\text{\%}}$. The left and right kidney doses were evaluated using the $D_{\text{mean}}$, $V_{6\text{\%}}$, and $V_{20\text{\%}}$. Lastly, the spinal canal was evaluated using the $D_{0.05\;{\text{cm}}^{3}}$.

Statistical significance between the strategies was determined using the Wilcoxon signed-rank tests. The analysis employs boxplots displaying median and interquartile range values, calculated across all patients.

## 3 Results

### 3.1 Evaluation of ITV coverage

Fig. 1 displays ITV coverage for an example patient with 7 mm margins. This figure reflects the overall behaviour of the strategies across all patients. The $D_{\text{98\%}}$ dose levels for all patients are provided in Fig. 2, with corresponding significance levels detailed in Table 1.

For non-adapted plans, ITV coverage fell below the replanning threshold for 18% of patients when plans were robustly optimized with 7 mm margins. When 2 mm margins were used, 47% of the patients required manual replanning. In the worst-case scenarios, these percentages increased to 41% and 83%, respectively.

Manually adapted MCA plans exhibited sufficient ITV coverage, all exceeding the specified threshold level. This applied to both 7 mm and 2 mm robust optimization margins, as well as to nominal and worst-case scenarios. These findings served as the baseline for the OAPT strategies.

Upon implementing ADR, the percentage of patients requiring manual replanning decreased to 6% when plans were robustly optimized for 7 mm, and to 18% for 2 mm margins. In the worst-case scenarios, these percentages shifted to 24% and 39%, respectively. Moreover, some outliers fell well below the $D_{\text{98\%}}$ threshold. These were the same patients with the lowest values in the non-adapted plans, indicating that ADR still provided an improvement.

AFA provided the largest improvement in ITV coverage. When the AFA strategy was applied using 7 mm margins, not a single patient required manual replanning. For robust optimization with 2 mm margins, 11% of patients required manual replanning. For the worst-case scenarios with 7 mm and 2 mm, the adaptation rates were 12% and 28%, respectively

### 3.2 Comparison of OAPT findings

For plans with 2 mm margins, the Wilcoxon signed-rank test indicated a significant improvement in ITV coverage when transitioning from the non-adapted plans to ADR, and further improvement when AFA was applied. This was observed for both the nominal and worst-case scenarios. For the 7 mm plans, there was no significant difference when ADR was applied compared to no adaptation in the nominal scenario. A significant improvement was observed with AFA, which performed on par with manually adapted MCA plans.

### 3.3 OAR exposure

The metrics for the heart, lungs, liver, and spinal canal remained within the targets derived from the PROTECT protocol. We observed only minor differences between the MCA and OAPT plans for these OARs. The results for all examined OAR metrics are presented in the Supplementary Figure S1 and S2.

The body dose at $0.05\;{\text{cm}}^{3}$ exceeded the threshold level for several patients, for both 7 mm and 2 mm margins. ADR exhibited worsening compared to the manually adapted plans. When AFA was applied, the results were not significantly worse compared to the manually adapted plan. The $D_{0.05\;{\text{cm}}^{3}}$ metric results of the body are displayed in Fig. 3. Notably, the most pronounced outliers were observed in patients who needed replanning due to inadequate ITV coverage.

For the left kidney, both the $D_{\text{mean}}$ and the $V_{6\text{\%}}$ metric were exceeded for some patients. This was most prevalent for ADR, although the differences between ADR and MCA were not large. This held true for both the 7 mm and 2 mm margins. The right kidney’s $V_{6\text{\%}}$ metric was exceeded for one patent using MCA and 7 mm margins, the findings are depicted in Fig. 3.

## 4 Discussion

We demonstrated that OAPT has the potential to reduce the need for manual replanning in oesophageal cancer treatment through the automated strategies ADR and AFA. AFA consistently outperformed ADR with respect to target coverage. These strategies enable the reduction of residual margins while maintaining manageable levels of manual replanning.

Reducing the need for manual replanning is desirable, as 18% of patients fell below the threshold with conventional 7 mm margins. ADR decreased this need substantially, while AFA proved adequate for all nominal plans. In worst-case scenarios, ADR cut the requirement for manual interventions by nearly half, and AFA further reduced it to almost one-fourth. Hence, full automation with minimal supervision seems accessible with the proposed tools. Residual margins of 2 mm proved unmanageable in a non-adaptive approach, and the proposed OAPT strategies could not eliminate the need for expert oversight. However, shifting from conventional non-adaptive 7 mm margins to OAPT allowed margin reduction. Implementing ADR successfully decreased margins to 2 mm, without increasing the need for manual replanning, while AFA provided even better results. Although these results highlight the potential for margin reduction, the optimal margin remains uncertain. Of the proposed automated OAPT strategies, the AFA method demonstrated the best results. However, full automation in clinical settings is currently not feasible with AFA, as it requires contour verification by the physician [25]. Conversely, ADR does not require this verification.

**Table 1:** Wilcoxon signed-rank test results for ITV coverage ($D_{98\text{\%}}$).

		**7 mm nominal**			**7 mm worst-case**		
		**MCA**	**AFA**	**ADR**	**MCA**	**AFA**	**ADR**

**NA**		<0.001	<0.001	n.s.	<0.001	<0.001	<0.05
**ADR**		<0.001	<0.001		<0.001	<0.001	
**AFA**		n.s.			n.s.		

		**2 mm nominal**			**2 mm worst-case**		
		**MCA**	**AFA**	**ADR**	**MCA**	**AFA**	**ADR**

**NA**		<0.001	<0.05	<0.001	<0.001	<0.001	<0.001
**ADR**		<0.001	<0.001		<0.001	<0.05	
**AFA**		<0.05			<0.001		

n.s. = Statistically non-significant result, where the p-value is ≥0.05.

The findings regarding OAR exposure and the associated clinical goals indicated that increased toxicities may occur when the proposed OAPT strategies were used [38]. For some patients, ADR results showed a significantly higher body dose and left kidney dose compared to manually adapted plans. While this study primarily focused on ITV coverage, the limited assessment of OARs underscores the need for caution, as improved target coverage may inadvertently increase treatment toxicity [5], [6], [7], [8], [9].

The effectiveness of OAPT strategies applied four weeks after treatment initiation was evaluated. Treatments usually consist of multiple fractions, with inter-fractional intervals considerably shorter than those examined in this study. While the proposed OAPT strategies address inter-fractional changes, organ motion on a much smaller time scale may still occur. These intra-fractional motions can cause dose distortions that cannot be dynamically incorporated into OAPT strategies, necessitating the use of robust optimization margins. Oesophageal motion during free breathing can be substantial [41], and breathing motion can also interfere with scanning motions, leading to interplay effects that introduce additional uncertainties [42]. The influence of cardiac motion on the oesophagus has been found to be negligible for proton therapy [43].

This research mainly focused on anatomical changes. It should be highlighted that the re-optimization itself is not fine-tuned. The treatment plans were created using a predetermined beam configuration consisting of one posterior beam supplemented by one or two posterior oblique beams. However, alternative beam configurations may be more suitable depending on the tumour’s location relative to the heart, as this can affect tumour coverage and OAR dosage [44].

Strategies based on Machine Learning (ML) have been proposed as alternatives to contour deformation methods. A comprehensive comparison of autocontouring methods for treatment targets demonstrated that deformable registration based contouring is not outperformed by ML based autocontouring for OAPT [45]. A combined autodelineation approach using contour deformation for the treatment target and specific OARs, along with ML for the majority of OARs, has proven to be the most effective method for autodelineation in oesophageal cancer [32]. Combining these approaches in OAPT methods for oesophageal cancer should be further investigated. A key factor in evaluating the effectiveness of the proposed OAPT strategies is the accuracy of the warp. Visual inspection suggests that these techniques achieve near-perfect alignment. However, the absence of a quantitative analysis to measure the algorithms’ effectiveness represents a limitation of this study.

The feasibility of the proposed OAPT techniques depends on achieving acceptable plan adaptation within an appropriate time frame. ADR in head and neck cancer cases typically takes 17.4 min [46], AFA requires longer computational time due to its reliance on MI-based deformable registration. Given treatment time constraints, these time spans are impractical. More research is needed to assess and minimize computational time for the proposed OAPT techniques.

The proposed OAPT strategies have demonstrated effectiveness with high quality CT images. While an in-room CT provides the desired image quality, it is not available in most clinical settings [47]. Typically, a Cone Beam Computed Tomography (CBCT) is used instead, but further research is needed to evaluate whether current CBCT quality is adequate for ADR and AFA. Additionally, CBCT acquisition times will remain lengthy due to the rotational speed limitations of the open architecture.

In conclusion, the proposed OAPT strategies for oesophageal cancer demonstrated a marked improvement over non-adaptive approaches. ADR and AFA could significantly reduce the need for manual replanning, thereby minimizing treatment postponements and alleviating workload. Additionally, these strategies contributed to margin reduction, enhancing dose conformity. Nonetheless, further research is needed to better understand the effects on OARs. The clinical implementation of OAPT is only feasible if the process is fast and fully automated. Addressing these challenges is essential for the effective integration of these strategies into practice.

## CRediT authorship contribution statement

**Pascal Herbst:** Conceptualization, Formal analysis, Investigation, Methodology, Writing – original draft. **Camille Draguet:** Methodology, Investigation, Writing – review & editing. **Ana M. Barragán-Montero:** Methodology, Investigation, Writing – review & editing. **Elena Borderías Villarroel:** Methodology, Investigation, Writing – review & editing. **Macarena Chocan Vera:** Investigation, Writing – review & editing. **Pieter Populaire:** Investigation, Writing – review & editing. **Karin Haustermans:** Project administration, Supervision, Writing – review & editing. **Edmond Sterpin:** Conceptualization, Methodology, Project administration, Supervision, Writing – review & editing.

## Declaration of competing interest

The authors declare the following financial interests/personal relationships which may be considered as potential competing interests: Pascal Herbst reports full-time employment at RaySearch Laboratories as Application Specialist from the 5 th of March 2024. Edmond Sterpin reports a relationship with RaySearch Laboratories Research Group that includes: non-financial support. Edmond Sterpin reports a relationship with IBA SA that includes: funding grants and non-financial support.

## Appendix A Supplementary data

MMC S1 Supplementary material.
