# Supplementary material for: Potential of automated online adaptive proton therapy to reduce margins for oesophageal cancer
Source: Phys Imaging Radiat Oncol. 2025 Jan 31;33:100712. doi: 10.1016/j.phro.2025.100712 (PMC11926429; doi:10.1016/j.phro.2025.100712)
Supplement: MMC S1 — Supplementary material. [file mmc1.pdf]

Supplementary Table S1. Clinical goals derived from the PROTECT protocol for oesophageal cancer.

Region of interest	Clinical goal
ITV D_{mean}	Between 49.39 Gy (RBE) and 51.40 Gy (RBE)
Body $D_{0.05\ \mathrm{cm}^3}$	At most 55.44 Gy (RBE)
Body $D_{1\ \mathrm{cm}^3}$	At most 53.98 Gy (RBE)
Lung D_{mean}	At most 20.00 Gy (RBE)
Lung $V_{20\%}$	At most 35% volume at 20.00 Gy (RBE)
Lung $V_{5\%}$	At most 70% volume at 5.00 Gy (RBE)
Spinal canal $D_{0.05\ \mathrm{cm}^3}$	At most 45.00 Gy (RBE) at 0.05 cm^3
Spinal canal (0.3 mm) $D_{0.05\ \mathrm{cm}^3}$	At most 50.00 Gy (RBE) at 0.05 cm^3
Liver $V_{30\%}$	At most 33% volume at 30.00 Gy (RBE)
Heart D_{mean}	At most 26.00 Gy (RBE)
Heart $V_{25\%}$	At most 50% volume at 25.00 Gy (RBE)
Heart $V_{40\%}$	At most 30% volume at 40.00 Gy (RBE)
Kidney left D_{mean}	At most 10.00 Gy (RBE)
Kidney left $V_{6\%}$	At most 30% volume at 6.00 Gy (RBE)
Kidney left $V_{20\%}$	At most 32% volume at 20.00 Gy (RBE)
Kidney right D_{mean}	At most 10.00 Gy (RBE)
Kidney right $V_{6\%}$	At most 30% volume at 6.00 Gy (RBE)
Kidney right $V_{20\%}$	At most 32% volume at 20.00 Gy (RBE)

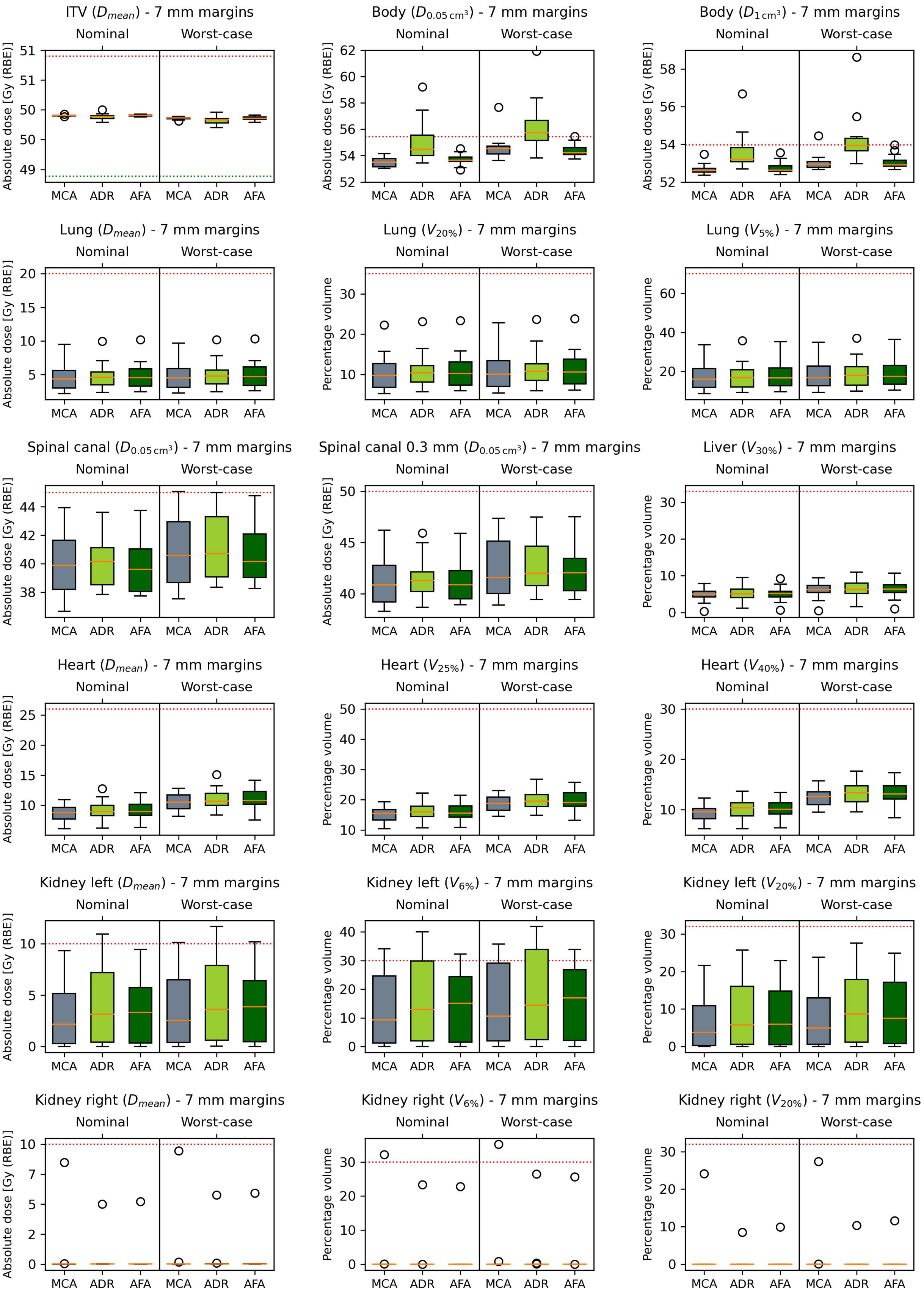

Supplementary Figure S1. OAR analysis employing 7 mm residual margins.

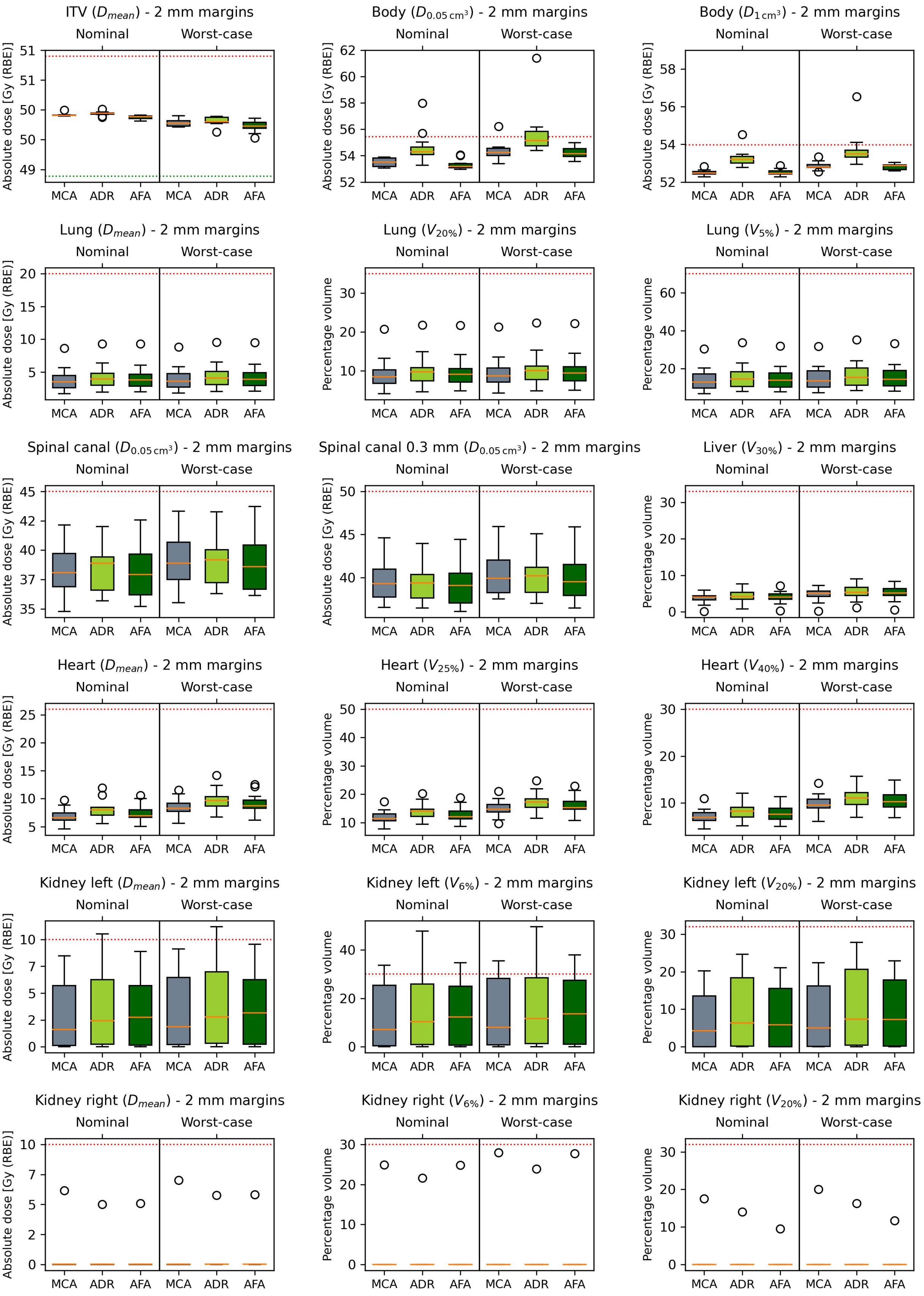

Supplementary Figure S2. OAR analysis employing 2 mm residual margins.
